# COVID-positive ankle fracture patients are at increased odds of perioperative surgical complications following open reduction internal fixation surgery

**DOI:** 10.1371/journal.pone.0262115

**Published:** 2021-12-31

**Authors:** Michael R. Mercier, Anoop R. Galivanche, Jordan P. Brand, Neil Pathak, Michael J. Medvecky, Arya G. Varthi, Lee E. Rubin, Jonathan N. Grauer

**Affiliations:** Department of Orthopaedics and Rehabilitation, Yale School of Medicine, New Haven, CT, United States of America; Medical College of Wisconsin, UNITED STATES

## Abstract

**Introduction:**

Ankle fractures have continued to occur through the COVID pandemic and, regardless of patient COVID status, often need operative intervention for optimizing long-term outcomes. For healthcare optimization, patient counseling, and care planning, understanding if COVID-positive patients undergoing ankle fracture surgery are at increased risk for perioperative adverse outcomes is of interest.

**Methods:**

The COVID-19 Research Database contains recent United States aggregated insurance claims. Patients who underwent ankle fracture surgery from April 1st, 2020 to June 15th, 2020 were identified. COVID status was identified by ICD coding. Demographics, comorbidities, and postoperative complications were extracted based on administrative data. COVID-positive versus negative patients were compared with univariate analyses. Propensity-score matching was done on the basis of age, sex, and comorbidities. Multivariate regression was then performed to identify risk factors independently associated with the occurrence of 30-day postoperative adverse events.

**Results:**

In total, 9,835 patients undergoing ankle fracture surgery were identified, of which 57 (0.58%) were COVID-positive. COVID-positive ankle fracture patients demonstrated a higher prevalence of comorbidities, including: chronic kidney disease, diabetes, hypertension, and obesity (p<0.05 for each). After propensity matching and controlling for all preoperative variables, multivariate analysis found that COVID-positive patients were at increased risk of any adverse event (odds ratio [OR] = 3.89, p = 0.002), a serious adverse event (OR = 5.48, p = 0.002), and a minor adverse event (OR = 3.10, p = 0.021).

**Discussion:**

COVID-positive patients will continue to present with ankle fractures requiring operative intervention. Even after propensity matching and controlling for patient factors, COVID-positive patients were found to be at increased risk of 30-day perioperative adverse events. Not only do treatment teams need to be protected from the transmission of COVID in such situations, but the increased incidence of perioperative adverse events needs to be considered.

## Introduction

While many ankle fractures can be treated nonoperatively, those with displacement and instability are typically considered for operative intervention (open reduction and internal fixation [ORIF]) [1]. Operative ankle fractures have continued to occur through the COVID pandemic and, regardless of patient COVID status, may need operative intervention for optimizing long term outcomes. The perioperative outcomes of COVID-positive patients undergoing ankle fracture ORIF has not been well defined.

Estimates of COVID positivity rates have varied widely throughout the pandemic, with reported peak seroprevalences ranging from 3.8% to 13.6% depending on geographic region [2–6]. Furthermore, the incidence of ankle fractures has previously been quoted to be 187 per 100,000 people each year [7]. Together, these numbers highlight that there were, and will continue to be, COVID-positive patients with ankle fractures.

Indicating patients for surgery is a process that balances risks and benefits. In general, ankle fracture surgery carries relatively low morbidity and is routinely performed for certain fractures types [8]. In addition to general anesthetic and operative ORIF risks, patients with COVID are thought to be at increased risk for perioperative adverse events above and beyond comorbidities that may have predisposed them to COVID [8, 9]. This is analogous to what was found with SARS-Cov-2 [10]. Nonetheless, the impact of COVID positivity on the clinical course of ankle ORIF patients remains unclear.

Defining the perioperative risks that COVID adds to operative interventions such as ankle fracture ORIF can be challenging due to statistical power. National databases have increasingly been used in the orthopedic literature to address such limitations [11–14]. However, most national databases have a year or more delay from data collection to release. For example, National Surgical Quality Improvement Program data releases in the fall after the data collection year [15]. Similarly, the National Inpatient Sample data releases data two years after the data collection year [16]. These delayed data releases can make current real-world data (RWD) difficult to obtain. To circumvent these limitations, the Symphony subsection of the Datavant COVID Research Database, which is a pro-bono, cross-industry collaboration was utilized to aggregate RWD year-to-date.

The first aim of the present study was to investigate prevalence of COVID in a population of patients undergoing ankle fracture ORIF in the above-noted Datavant COVID Research Database at the start of the pandemic. The second aim of the study was to assess the impact of COVID on 30-day perioperative outcomes of those with versus without COVID. We hypothesized that COVID-positivity is associated with greater risk of postoperative adverse events in patients undergoing surgery for urgent orthopedic indications, such as ankle fracture.

## Methods

### Data source

A retrospective cohort analysis was performed based on the Symphony Healthcare data subset of the Datavant COVID-19 Research Database. The date range evaluated was April 1^st^, 2020 (the effective start date of the COVID ICD-10 code) and July 15^th^,2020. This time frame captured the start of the COVID pandemic. The current study was determined exempt from review by our institution’s Intuitional Review Board.

Access to the Datavant COVID-19 Research Database was granted by the database’s Scientific Steering Committee after approval of our research group’s hypothesis-driven research proposal. The COVID-19 Research Database is a secure repository of HIPAA-compliant datasets which aggregates longitudinal patient-level data from a large consortium of healthcare institutions and organizations [17].

The Symphony Healthcare data subset was utilized for the current study. This encompasses data on over 280 million patients, 1.8 million prescribers, and 16,000 health plans [18]. Patient-level data was extracted from medical insurance claims in the dataset, which provided information on demographics, comorbidities, and perioperative adverse events.

### Study population

Patients were first selected by a diagnosis of ankle fracture (International Classification of Diseases, 10th Revision [ICD-10] codes S82.0–S82.9). From the identified group, only patients with Current Procedural Terminology (CPT) codes 27766 (ORIF of medial malleolus fracture), 27769 (ORIF of posterior malleolus fracture), 27792 (ORIF of lateral malleolus fracture), 27814 (ORIF of bimalleollar fracture), and 27822/27823 (ORIF of trimalleollar fracture) were included in our analysis.

COVID-positivity was identified by presence of an emergency ICD-10 COVID code (U07.1) anytime from the date of surgery back fourteen days prior to surgery. It is noted that some patients with COVID may not have been diagnosed and received this code, but it was believed that the number of COVID patients in the COVID-negative group would be sufficiently low to not affect study results.

### Patient data

Demographic variables extracted and assessed from the database included patient age and sex. Comorbidities were defined based on ICD-10 codes (codes presented in S1 Table). Comorbidities assessed included: asthma, chronic kidney disease, congestive heart failure, chronic obstructive pulmonary disease, coronary artery disease, diabetes, hypertension, and obesity. A one-year retrospective look-back period was used to collect comorbidities data.

Thirty-day postoperative adverse events were also identified based on ICD-10 codes (codes presented in S2 Table). Perioperative outcomes of interest were aggregated into any, major, and minor adverse events. Serious adverse events included: surgical sites infection, sepsis, venous thromboembolism, deep vein thrombosis, cardiac arrest, myocardial infarction, and pancreatitis. Minor adverse events included: pneumonia, urinary tract infection, acute kidney injury, and wound dehiscence. Any adverse event was counted when a patient experienced a major or minor adverse event.

### Statistical analysis

Patient demographics, comorbidities, and adverse events data were compared between COVID-positive patients and COVID-negative patients. Categorical demographic and adverse events were compared using chi-squared tests. Any, major, and minor postoperative adverse events were also compared using a chi-squared test.

Propensity-score matching on the basis of age, sex, and all examined comorbidities was utilized to compare the COVID-positive cohort versus a 1:10 matched COVID-negative cohort in both univariate and multivariate analyses. A multivariate regression was performed on the combined COVID-positive and 1:10 matched COVID-negative population to assess the effect of demographic and comorbidity variables on patients’ likelihood of experiencing any postoperative adverse event.

Patient data was initially queried from the COVID-19 Research Database using Snowflake (Snowflake, San Mateo, CA), and all subsequent data processing was performed in R (R Core Team, 2020).

## Results

### Patient population

In total, 9,805 ankle fracture ORIF patients met criteria for inclusion in the patient sample. 57 patients (0.58%) tested positive for COVID in the two weeks prior to surgery (Table 1). COVID-positive patients were similar to unaffected patients in age (mean +/- standard deviation of 49.88 +/- 17.99 years versus 49.25 +/- 18.33, p = 0.793), sex (28.07% vs. 36.72% female, p = 0.211), and procedure performed (p = 0.206).

**Table 1:** Demographic and comorbid characteristics of ankle fracture patients by COVID diagnosis.

	COVID (–)		COVID (+)			1:10 Matched COVID (–) Cohort		
	Number	Percent	Number	Percent	*p-value	Number	Percent	P-Value
Total Patients = 9,835	9,778	99.42%	57	0.58%		570	5.48%	
**Age**								
0–19.9	616	6.30%	3	5.26%	0.984	41	7.19%	0.952
20–39.9	2,501	25.58%	15	26.32%		154	27.02%	
40–59.9	3,266	33.40%	20	35.09%		190	33.33%	
60 +	3,325	34.00%	19	33.33%		185	32.46%	
**Sex**								
Male	3,590	36.72%	16	28.07%	0.211	141	24.74%	0.694
Female	6,118	62.57%	41	71.93%		429	75.26%	
**Procedure Type (CPT Code)**								
ORIF of bimalleollar fracture (27814)	3,154	32.26%	20	35.09%	0.206	38	6.67%	0.103
ORIF of lateral malleolus fracture (27792)	2,992	30.60%	13	22.81%		0	0.00%	
ORIF of trimalleolar fracture (27822)	2,149	21.98%	17	29.82%		176	30.88%	
ORIF of medial malleolus fracture (27766)	733	7.50%	3	5.26%		182	31.93%	
ORIF of trimalleolar fracture (27823)	646	6.61%	2	3.51%		136	23.86%	
ORIF of posterior malleolus fracture (27769)	104	1.06%	2	3.51%		38	6.67%	

* = Statistically significant at p < 0.05.

Comorbidity characteristics are presented in Table 2. These were first compared between the two patient cohorts using univariate chi-squared tests. COVID-positive patients were more likely to have chronic kidney disease (14.04% versus 5.98%, p = 0.024), diabetes (29.82% versus 15.18%, p = 0.004), hypertension (29.82% versus 15.10%, p = 0.010), and obesity (36.84% versus 19.92%, p = 0.003).

**Table 2:** Comorbidity characteristics of patients by COVID status.

	COVID (–)		COVID (+)			1:10 Matched COVID (–) Cohort		
	Number	Percent	Number	Percent	*p-value	Number	Percent	*p-value
Total Patients = 9,835	9,778	99.42%	57	0.58%		570	50.00%	
Asthma	889	9.09%	9	15.79%	0.129	106	18.60%	0.732
**Chronic Kidney Disease**	**585**	**5.98%**	**8**	**14.04%**	**0.024**	70	12.28%	0.863
Congestive Heart Failure	469	4.80%	5	8.77%	0.277	36	6.32%	0.664
COPD	645	6.60%	7	12.28%	0.146	**54**	9.47%	0.655
Coronary Artery Disease	718	7.34%	8	14.04%	0.094	69	12.11%	0.832
**Diabetes**	**1,476**	**15.10%**	**17**	**29.82%**	**0.004**	171	30.00%	1.000
**Hypertension**	**3,456**	**35.34%**	**30**	**52.63%**	**0.010**	324	56.84%	0.638
**Obesity**	**1,948**	**19.92%**	**21**	**36.84%**	**0.003**	202	35.44%	0.947

* = Statistically significant at p < 0.05.

A 1:10 COVID-negative cohort was constructed and propensity matched on the basis of age, sex, and comorbidities for comparison to the COVID-positive cohort. After propensity matching, no statistically significant differences between demographic or comorbidity factors were noted between the COVID-positive and negative groups (Tables 1 and 2).

### Adverse events

Adverse events occurring within the 30-day postoperative period were tabulated and compared by univariate analyses for the full groups and propensity matched groups and multivariate for the propensity matched groups (Table 3). For the full groups, based on univariate analyses, COVID-positive patients had higher rates of any adverse events (17.54% compared with 6.04%, p<0.001), serious adverse events (8.77% compared with 2.60%, p = 0.013), and minor adverse events (14.04% compared with 4.47%, p = 0.002). With regards to specific complications, COVID-positive patients had higher rates of sepsis (7.02% compared with 0.61%, p<0.001), pneumonia (7.02% compared with 0.71%, p<0.001), and acute kidney injuries (8.77% compared with 1.87%, p<0.001).

**Table 3:** Adverse events breakdown by COVID status.

Complication	COVID (–)		COVID (+)		P-Value	1:10 Matched COVID (–) Cohort		P-Value	Propensity Matched Multivariate Odds Ratio §		
Total Patients = 9,835	9,778	99.42%	57	0.58%		570	5.48%		OR	95% CI	p-value
**Any Adverse Event (AAE)**	**591**	**6.04%**	**0**	**17.54%**	**<0.001**	**44**	**7.72%**	**0.007**	**3.89**	**[1.63–8.85]**	**0.002**
**Serious Adverse Event (SAE)**	**254**	**2.60%**	**5**	**8.77%**	**0.013**	**15**	**2.63%**	**0.006**	**5.48**	**[1.78–15.48]**	**0.002**
Surgical site infection	55	0.56%	0	0.00%	1.000	3	0.53%	1.000			
** Sepsis **	**60**	**0.61%**	**4**	**7.02%**	**<0.001**	**4**	0.70%	**<0.001**			
Pulmonary Embolism	127	1.30%	1	1.75%	1.000	8	1.40%	0.512			
Deep Vein Thrombosis	88	0.90%	0	0.00%	0.989	4	0.70%	1.000			
Cardiac Arrest	9	0.09%	1	1.75%	0.065	0	0.00%	0.154			
Myocardial Infarction	24	0.25%	0	0.00%	1.000	2	0.35%	1.000			
Pancreatitis	8	0.08%	0	0.00%	1.000	0	0.00%	1.000			
**Minor Adverse Event (MAE)**	**437**	**4.47%**	**8**	**14.04%**	**0.002**	38	6.67%	0.077	**3.10**	**[1.13–7.85]**	**0.021**
**Pneumonia**	**69**	**0.71%**	**4**	**7.02%**	**<0.001**	**8**	**1.40%**	**0.015**			
Urinary Tract Infection	149	1.52%	2	3.51%	0.500	21	3.68%	1.000			
** Acute Kidney Injury**	**183**	**1.87%**	**5**	**8.77%**	**<0.001**	**13**	**2.28%**	**0.017**			
Wound Dehiscence	102	1.04%	0	0.00%	0.905	7	0.07%	0.857			

§ 1:1 Matching on age, sex, and all comorbidities. Bolding indicates statistic.

For the propensity matched groups, based on univariate analyses, statistical significance was maintained when comparing COVID-positive patients to the 1:10 COVID-negative cohort. COVID-positive patients had higher rates of any adverse events (17.54% compared with 7.72%, p = 0.007, serious adverse events (8.77% compared with 2.63%, p = 0.006), and minor adverse events (14.04% compared with 6.67%, p = 0.077). With regards to specific complications, COVID-positive patients had higher rates of sepsis (7.02% compared with 0.70%, p<0.001), pneumonia (7.02% compared with 1.40%, p = 0.015), and acute kidney injuries (8.77% compared with 2.28%, p = 0.017).

**Fig 1:**
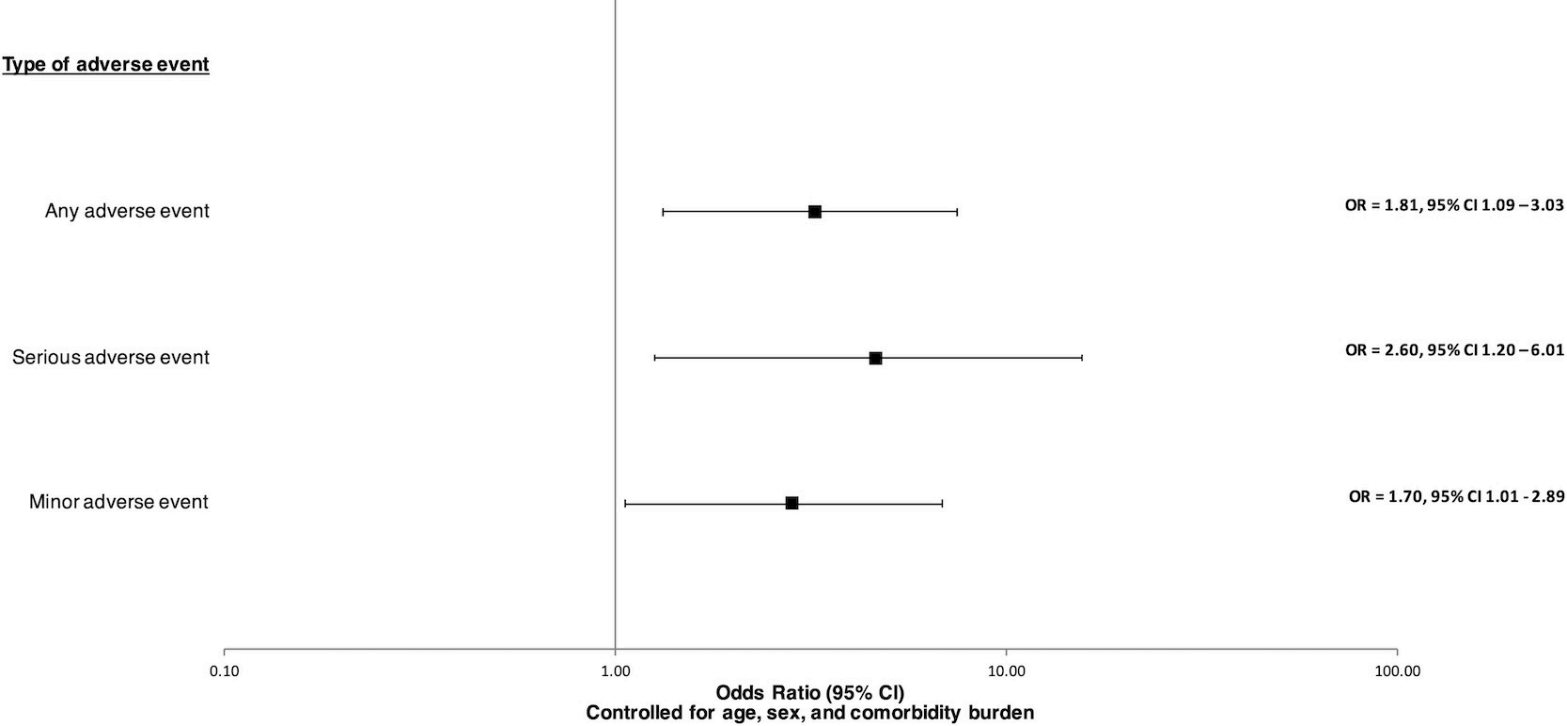
Propensity score matched multivariate odds ratios for adverse events after ankle fracture surgery in COVID (+) patients.

A multivariate regression analysis was then performed controlling for patient-specific differences in age, sex, and comorbidities (Table 3, Fig 1). Based on the relatively low numbers of adverse events, this was just done for the aggerated adverse event categories. The COVID-positive cohort were found to have increased odds of any adverse events (OR = 3.89; 95% CI, 1.63–8.85; p = 0.002), serious adverse events (OR = 5.48; 95% CI, 1.78–15.48; p = 0.002), and minor adverse events (OR = 3.10; 95% CI, 1.13–7.85; p = 0.021).

## Discussion

Since being declared a pandemic by the World Health Organization in March of 2020, COVID has continued to place an unprecedented degree of stress on healthcare systems worldwide. Currently, the pathophysiologic mechanisms through which COVID mediates disease is not entirely clear [19]. In surgery, little is known regarding the effects of surgery or anesthesia on the postoperative course of COVID patients [20]. Prior studies thus far that have examined postoperative complication risk among COVID patients in orthopedics have been conducted using single institution samples, with limited statistical power [21]. To our knowledge, the current study is the first of its kind to utilize a large patient sample to find that COVID-positive patients are at significantly elevated odds of adverse events following ankle fracture surgery compared to COVID-negative patients, even after accounting for individual patient demographic factors and comorbidities.

The overall COVID positivity rate for ankle fractures cases in our analysis was 0.58%, which was ascertained by the utilization of the emergency diagnostic code U07.1. Reported COVID-positivity rates have varied widely by patient demographic and geographic region [22–25]. The diagnostic code U07.1 is only utilized for patients who tested positive for COVID by laboratory testing, and was introduced on April 1^st^, 2020. Thus, the positivity rate reported in our study may be an underestimation of the true COVID-positivity rate among ankle fracture patients. However, as the rate of COVID-positive patients in the COVID-negative group would inherently be a very small percentage of the overall population, missing a low number of COVID-positive patients would not be expected to sway the result of the current study.

The current study demonstrates a significantly higher prevalence of several comorbidities among COVID-positive patients: chronic kidney disease, diabetes, hypertension, and obesity. This finding is consistent with prior studies that have demonstrated a similar comorbidity profile in the overall COVID patient population [26–28]. Additionally, the rate of comorbidities observed among COVID-negative patients in the current study is in accordance with prior ankle fracture cohort studies [29].

When controlling for patient demographics and overall comorbidity burden through a multivariate regression using propensity score matching, COVID-positivity was independently associated with a significantly increased odds of the occurrence of any, major, or minor adverse event following ankle ORIF. These findings are consistent with a prior international cohort study that demonstrated of certain complications among COVID-positive patients undergoing surgery [10].

In the univariate analysis of specific adverse events, the current study found that pneumonia was found to be higher in the COVID-positive ankle fracture cohort (~7% versus ~1%), which is particularly interesting and somewhat intuitive. A wealth of literature has also described COVID as a COVID as a disease of endothelial cell dysfunction promoting a pro-coagulative state [30–32]. Thus, one may expect a significantly higher rates of postoperative clinical events such as pulmonary embolism and deep vein thrombosis in the COVID-positive cohort. However, given the relatively low incidence of venous thromboembolic events in the current study, we are unable to further assess the relationship between COVID-positivity and thromboembolic events amongst ankle fracture surgery patients.

The current study has several limitations which must be considered when interpreting its results. First, the current study relies on medical insurance claims data, which is dependent on professional ICD coding (in particular, as discussed above, some cases of COVID may not have captured the code used for its identification). Additionally, claims information lacks data on severity and duration of patient comorbidities, and postoperative outcomes related to the operative procedure are not explicitly included in claims data. Secondly, the Symphony database is not a weighted sample, making it difficult to make nationally representative estimates of overall COVID-positivity. Thirdly, our ability to accurately determine orthopedic-specific postoperative endpoints of interest, such as degree of fracture reduction, range of motion, and later posttraumatic osteoarthritis, are limited given the nature of the database. Finally, the database also does not reliably capture mortality as an endpoint, making it difficult to estimate perioperative mortality risk among our patient population.

In conclusion, the current study found that 0.58% of ankle fracture surgery patients during the study period were positive for COVID. COVID-positivity conferred a significantly increased odds of adverse events postoperatively. This difference remained even after controlling for patient demographics and comorbidity burden. These findings suggest that surgery should be undertaken cautiously in patients who have or may have COVID, not just for the safety of providers, but also patients. As hospital environments continue to be exposed to COVID and there is the concern of further waves of the pandemic, care must be taken when treating COVID-positive ankle fracture patients.

## Supporting information

S1 Table List of examined comorbidities and the corresponding ICD-10 diagnostic codes used to define each. (DOCX)

S2 Table List of examined adverse events and the corresponding ICD-10 diagnostic codes used to define each. (DOCX)
