# Supplementary material for: COVID-positive ankle fracture patients are at increased odds of perioperative surgical complications following open reduction internal fixation surgery
Source: PLoS One. 2021 Dec 31;16(12):e0262115. doi: 10.1371/journal.pone.0262115 (PMC8719674; doi:10.1371/journal.pone.0262115)
Supplement: S1 Table — (DOCX) [file pone.0262115.s001.docx]

| **S1 Table. List of examined comorbidities and the corresponding ICD-10 diagnostic codes used to define each.** | |
| --- | --- |
| **Comorbidity** | **ICD-10 Diagnostic Code** |
| Asthma | J45 |
| Chronic Kidney Disease | N18 |
| Congestive Heart Failure | I50 |
| Chronic Obstructive Pulmonary Disease | J44 |
| Coronary Artery Disease | I25 |
| Diabetes | E10, E11, E12, E13, E14 |
| Hypertension | I10, I11, I12, I13, I15 |
| Obesity | E66 |
