# Supplementary material for: COVID-positive ankle fracture patients are at increased odds of perioperative surgical complications following open reduction internal fixation surgery
Source: PLoS One. 2021 Dec 31;16(12):e0262115. doi: 10.1371/journal.pone.0262115 (PMC8719674; doi:10.1371/journal.pone.0262115)
Supplement: S2 Table — (DOCX) [file pone.0262115.s002.docx]

| **S2 Table. List of examined adverse events and the corresponding ICD-10 diagnostic codes used to define each.** | |
| --- | --- |
| **Adverse Event** | **ICD-10 Diagnostic Code** |
| Surgical Site Infection | T81.40, T81.41, T81.42, T81.43, T81.49 |
| Sepsis | T81.44, T81.12, A41 |
| Pulmonary Embolism | I26 |
| Deep Vein Thrombosis | I82 |
| Cardiac Arrest | I46, I97.12 |
| Myocardial Infarction | I21 |
| Pancreatitis | K85 |
| Pneumonia | J13, J14, J15, J16, J17, J18 |
| Urinary Tract Infection | N39 |
| Acute Kidney Injury | N17 |
| Wound Dehiscence | T81.3 |
